# Fetal Cyclopia, Proboscis, Holoprosencephaly, and Polydactyly: A Case Report With Review of Literature

**DOI:** 10.7759/cureus.34576

**Published:** 2023-02-02

**Authors:** Raja Kollu, Sai Kotamraju, Seema Uligada, Mary Varunya

**Affiliations:** 1 Radiodiagnosis, Narayana Mallareddy hospital , Malla Reddy Medical College for Women, Hyderabad, IND; 2 Radiodiagnosis, Malla Reddy Medical College for Women, Hyderabad, IND; 3 Radiology, Vydehi Institute of Medical Sciences and Research Centre, Bengaluru, IND

**Keywords:** patau syndrome, trisomy 18, trisomy 13, ventricular septal defects, post-axial polydactyly, alobar holoprosencephaly, proboscis, cyclopia

## Abstract

We present a case of holoprosencephaly (HPE) with cyclopia and proboscis. The mother was a 35-year-old, G1P1 with no known comorbid conditions, not in a consanguineous marriage, and with no history of illicit drug use. On a routine antenatal ultrasound scan, features of alobar HPE, proboscis, and other anomalies were identified. The mother was counseled about the condition and as per their consent, the pregnancy was terminated. After labor induction, she gave birth to a female neonate weighing 1,000 g. The newborn's Apgar score could not be calculated. In the initial physical examination, an eye and a 3.5-cm proboscis were seen in the middle of the forehead. The newborn had no nose, and the outer ears were normal. On postmortem examination, alobar HPE, polydactyly, ventricular septal defect, and myelomeningocele were confirmed. This case report highlights the importance of attention to these details during antenatal scans for early detection in order to reduce the maternal and neonatal health burden.

The pictures presented in this article were taken after obtaining parental consent.

## Introduction

Holoprosencephaly refers to a group of disorders arising from failure of normal forebrain development, or incomplete cleavage of the prosencephalon, during embryonic life. The presence of a single eye or a partially divided eye in the same orbit, an absence of a nose, and a proboscis above the eye are characteristics of cyclopia. Polydactyly, renal dysplasia and cardiac anomalies such as ventricular septal defect have been reported in stillborns with this condition. An omphalocele-a saclike protrusion from the umbilicus - may also be present in cases with this disorder.

The disorder is rare and can be caused by various factors such as genetic mutations and exposure to teratogenic substances during pregnancy. In its worst form, cyclopia, fetuses do not reach full term and those that do, do not survive extra uterine life. The most commonly involved genetic syndrome with cyclopia is trisomy 13 (Patau syndrome) [1] and implicated genetic mutation being SHH (Sonic HedgeHog Gene Regulator) which is involved in the separation of the single eye field into two bilateral fields.

Patau syndrome is a clinical syndrome that occurs when all or some cells of the body contain an extra copy of chromosome 13. It was first described by Patau et al. in 1960 as a separate syndromic entity [2]. Trisomy 13 fetuses may show various anomalies including holoprosencephaly, omphalocele, kidney, and urogenital anomalies, hyperechogenic bowel, cardiac septal defects, single umbilical artery (SUA), myelomeningocele, radial aplasia, polydactyly, and flexion deformity of the fingers [3]. The key features are neurological impairment and facial dysmorphism.

There are several risk factors implicated in the causation of these disorders, but it is not uncommon to find no single risk factor like in the case described in this article. Since these anomalies are incompatible with life, early antenatal attendance and identification of such cases by ultrasonography are important parts of treatment planning as well as discussion of termination of pregnancy with parents when possible.

## Case presentation

A 35-year-old G1P1, with a single live intrauterine gestation, came for regular antenatal checkups and scans at 17 weeks of gestation with spontaneous conception. The mother had no known comorbid conditions, was not in a consanguineous marriage, and had no history of illicit drug use.

Radiological findings

**Figure 1:**
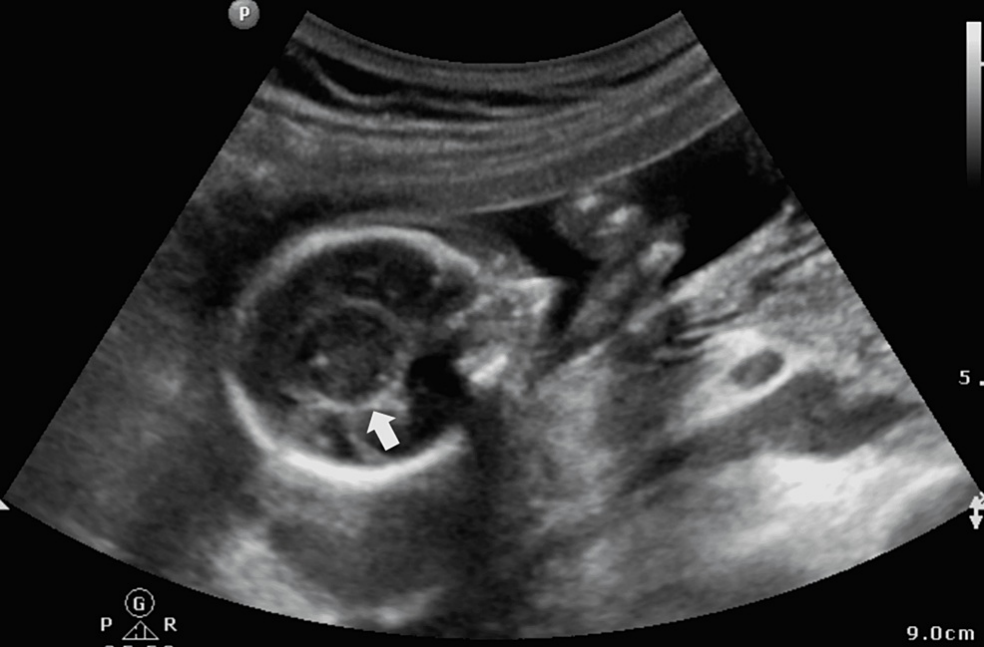
Fused thalami in mid-line (white arrow).

**Figure 2:**
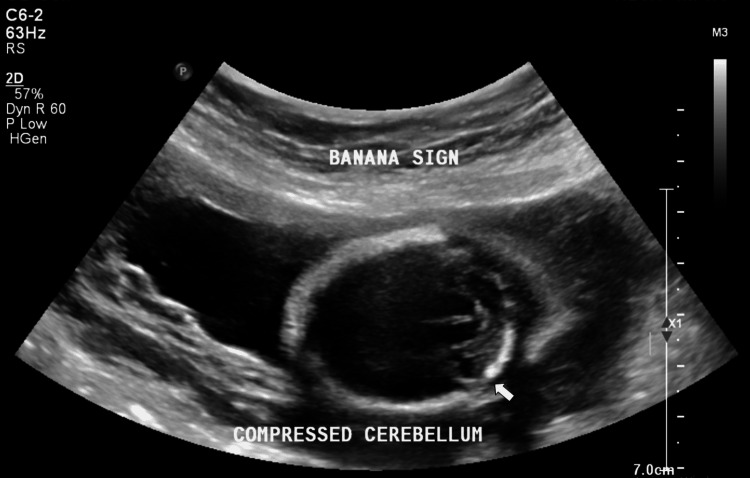
Posteriorly compressed cerebellum - Banana Sign (White arrow)

**Figure 3:**
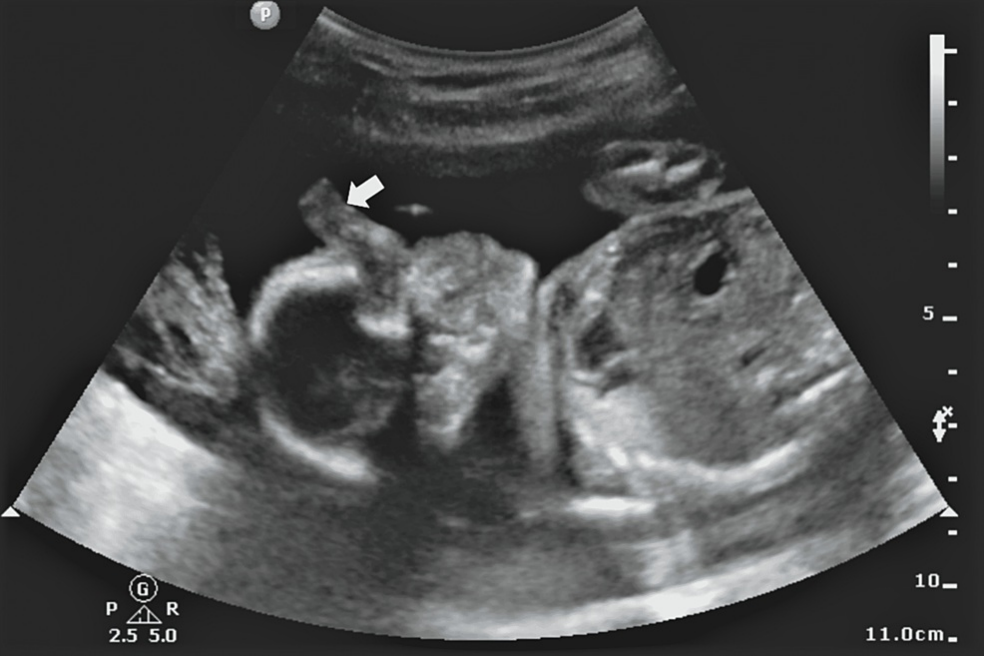
A snout-like protrusion from the forehead with absent nasal bone - Proboscis (white arrow)

**Figure 4:**
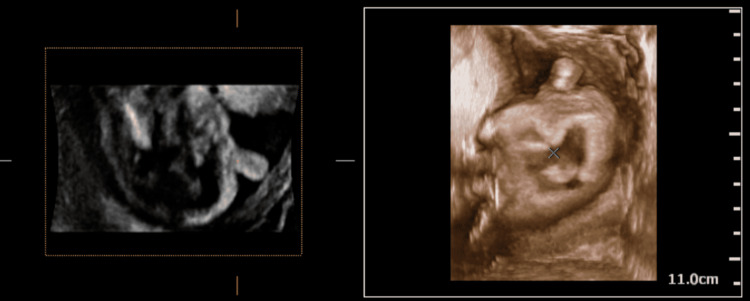
B-Mode image with corresponding VR image of the snout-like protrusion from the forehead with absent nasal bone - Proboscis VR: Volume Rendering

**Figure 5:**
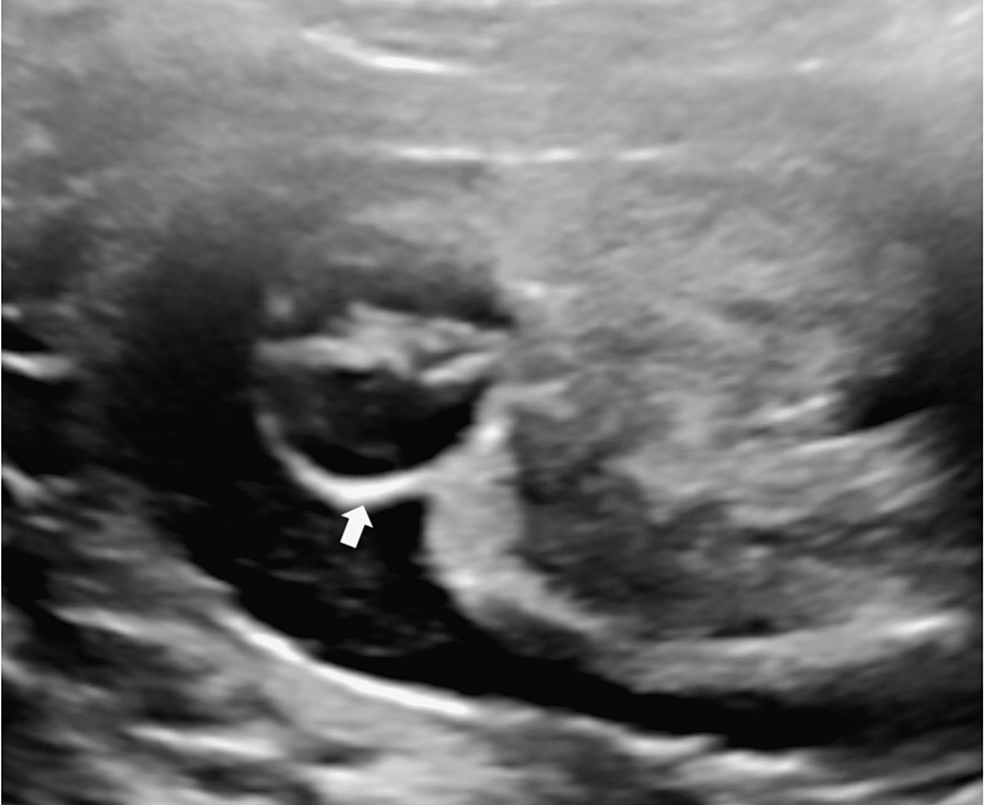
Open neural tube defect with splayed or divergent posterior elements at the sacral region - Myelomeningocele (White arrow)

**Figure 6:**
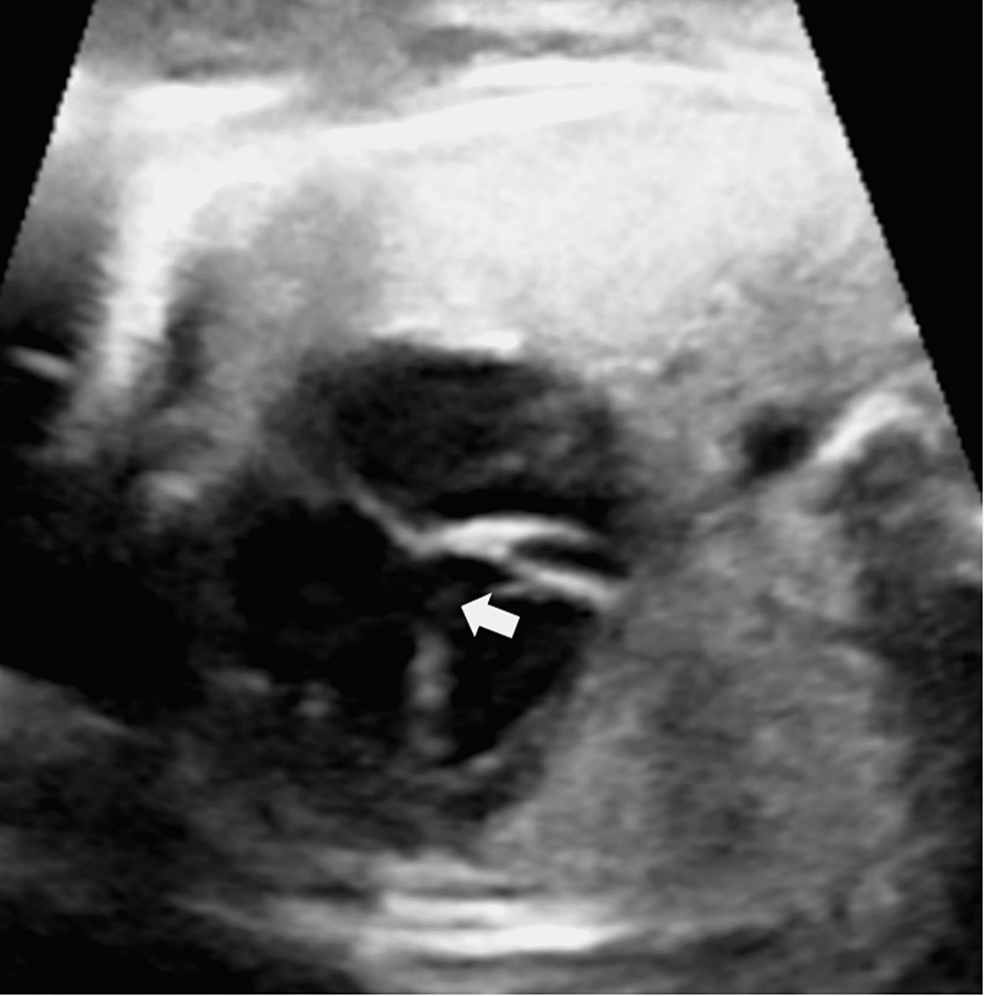
Cardiac interventricular septal defect (white arrow).

On B-Mode USG, the findings were an absent septum pellucidum with a single large ventricle. Bilateral thalami were fused in the midline (Figure 1). The cerebellum was compressed posteriorly to give the typical banana sign (Figure 2). On a longitudinal facial profile view, a snout-like protrusion from the forehead with absent nasal bone was noted (Figures 3, 4). Bilateral orbits were not visualized. Evidence of an open neural tube defect with splayed or divergent posterior elements at the sacral region was identified (Figure 5). An interventricular cardiac septal defect of 5 mm was also found (Figure 6).

On counseling the patient about the findings and their future implications, the pregnancy was terminated. Labor induction was done under the obstetrics and gynecology department supervision. A female stillborn neonate weighing 1000gms was vaginally delivered. The postpartum period of the mother was uneventful. 

Gross anatomical findings

**Figure 7:**
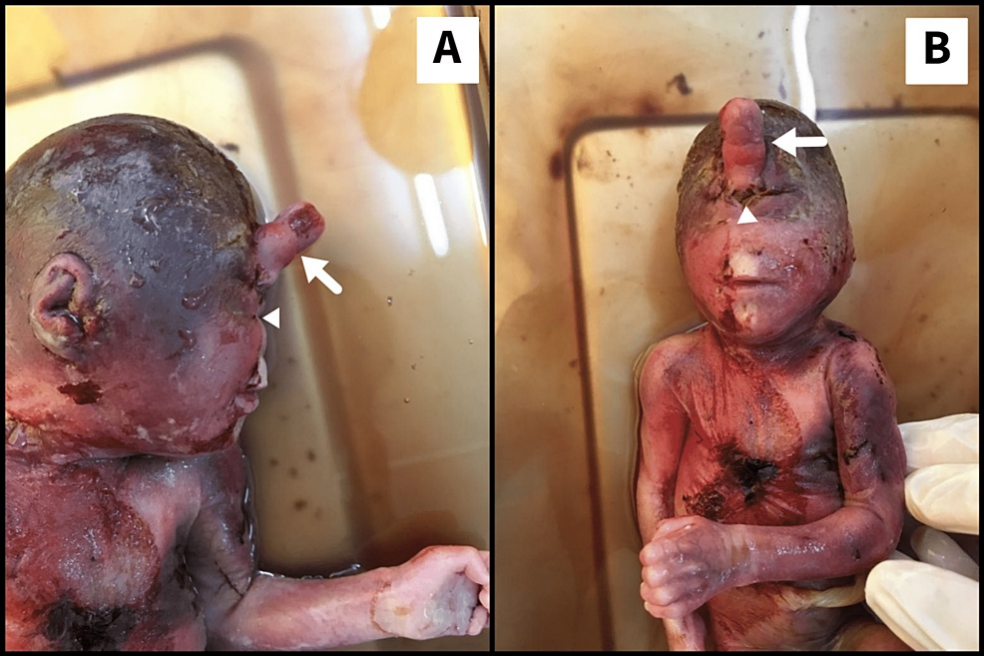
(A) Lateral profile view. (B) Frontal view. A snout-like projection from the forehead (white arrow) with a single suspicious palpebral fissure below the projection (white arrowhead) with no identifiable nose.

**Figure 8:**
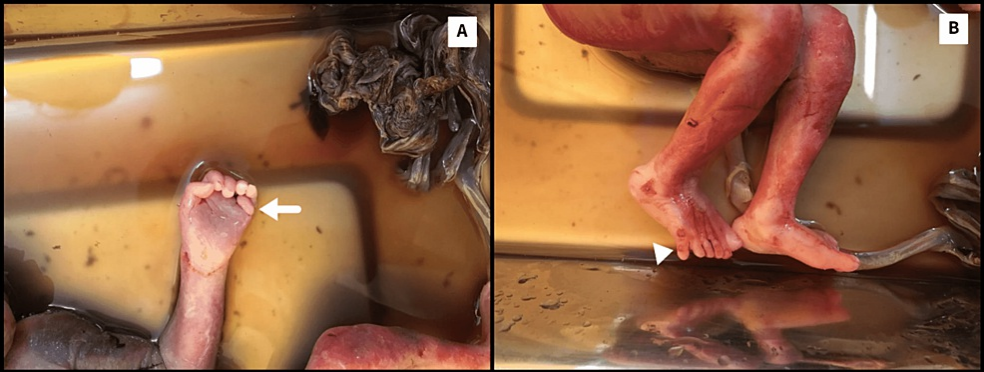
(A) Extra-axial digit in upper limb (white arrow). (B) Extra-axial digit in lower limb (white arrowhead).

**Figure 9:**
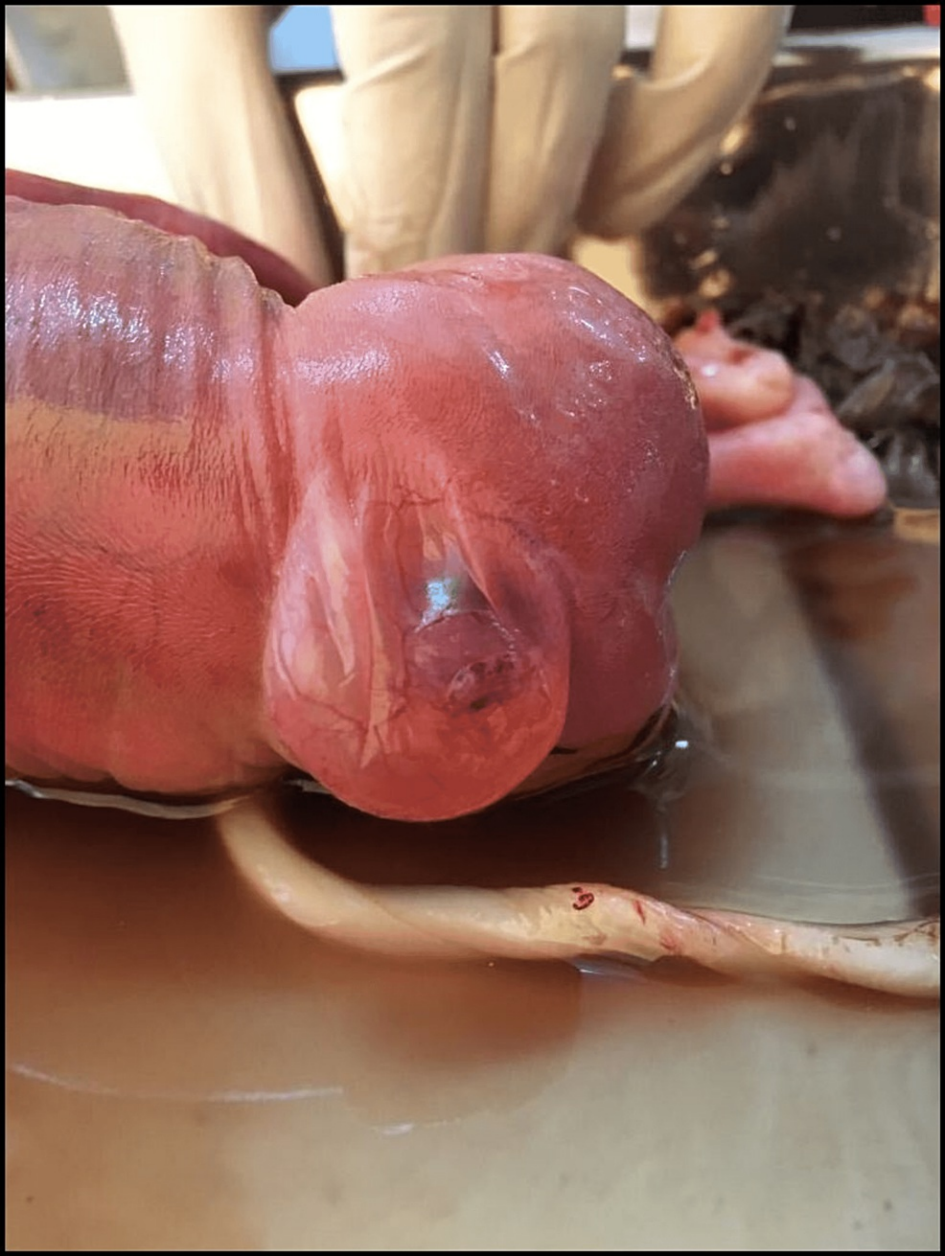
A subcutaneous cystic collection in the sacral region - Myelomeningocele.

The ultrasound findings were reconfirmed. A 3.5 cm snout-like projection from the forehead was identified with a single suspicious palpebral fissure below the projection (Figures 7A, 7B). No identifiable nose was noted. Additional findings of extra-axial digits were noted in bilateral upper and lower limbs (Figures 8A, 8B). A subcutaneous cystic collection was noted in the sacral region (Figure 9). Further genetic analysis of the fetus was not obtainable due to financial constraints.

## Discussion

HPE is a complex human brain malformation resulting from incomplete cleavage of the prosencephalon into the right and left hemispheres [4]. Three levels of increasing severity of HPE are described: lobar HPE, where the right and left ventricles are separated, but with some continuity across the frontal cortex; semi lobar HPE with a partial separation, and the most severe form, alobar HPE, with a single brain ventricle and no interhemispheric fissure [4]. A series of facial anomalies are frequently associated, owing to the common origin of the embryonic forebrain and mid-face from the prechordal mesoderm, along with some other anomalies [5]. Facial anomalies associated with alobar HPE are cyclopia and proboscis [4]. Small indicators such as polydactyly, ventricular septal defects, and myelomeningocele require keen attention to detail. This prenatal diagnosis often leads to the termination of pregnancy, after genetic counseling, regarding the severity of the malformations [4]. The survival rate is very low. The majority of cases are sporadic in origin although genetic etiology and the role of teratogens have also been attributed.

Syndromes associated with alobar HPE sequence

Trisomy 13

Trisomy 13, or Patau syndrome, is the most common cause of HPE [6]. Multiple organ systems can be affected in trisomy 13 and common physical exam findings include microphthalmia/anophthalmia, cleft lip and palate, postaxial polydactyly, and rocker bottom feet. HPE has been reported in 8%-39% of individuals with trisomy 13 [6]. Due to the high incidence of trisomy 13 in HPE cases, trisomy 13 is the top differential diagnosis for HPE. The mechanism of trisomy 13’s relationship to HPE is not clearly understood but ZIC2 gene variants, which are loss-of-function mutations, are considered major targets.

Other Aneuploidies

Trisomy 13 is the most common aneuploidy associated with HPE, with trisomies 18, 21 and 22 being the next most common. Central nervous system anomalies found in trisomy 18 include corpus callosum agenesis, spina bifida, and cerebellar hypoplasia. HPE is a less common CNS manifestation in trisomy 18 [7].

Smith-Lemli-Opitz Syndrome

Smith-Lemli-Opitz syndrome (SLOS) is a multiple congenital anomaly syndrome that presents with intellectual disability, facial dysmorphisms, congenital heart anomalies, and external genitalia defects in males.

Pseudotrisomy 13

Pseudotrisomy 13 syndrome, also known as HPE-polydactyly syndrome, refers to HPE associated with postaxial polydactyly and a normal karyotype. Because HPE and polydactyly are features of trisomy 13, the term pseudotrisomy 13 is given for this presentation with normal karyotype. 80% of cases with classic HPE (MRI evidence of HPE), 80% with polydactyly, and 58% with a cardiac anomaly present with this genotypic variant [8]. There is no known molecular diagnostic association and research with next generation sequencing is needed to more thoroughly interrogate pseudotrisomy 13 for a molecular diagnosis [7].

Pallister-Hall Syndrome

Pallister-Hall syndrome is the presence of a hypothalamic hamartoma and mesoaxial polydactyly, with heterozygous pathogenic variant in GLI3 [7]. Though the majority of cases result in termination of pregnancy, if not diagnosed early, it results in severe physical and psychological maternal and neonatal morbidity.

The limitations of the present case report are the unavailability of the genetic analysis reports to differentiate Trisomy 13 and pseudotrisomy 13.

## Conclusions

In order to properly counsel parents with fetal anomalies, prenatal assessment, including in-depth ultrasound and amniocentesis, is crucial. With careful attention to detail and appropriate advanced imaging, conditions such as HPE, polydactyly, and ventricular septal defects that may be part of various syndromes can be diagnosed with confidence at a very early stage.
